# Traumatic auricular hematoma

**DOI:** 10.11604/pamj.2017.26.148.11372

**Published:** 2017-03-15

**Authors:** Moncef Sellami, Abdelmonem Ghorbel

**Affiliations:** 1 Department of Otorhinolaryngology-Head and Neck Surgery Habib Bourguiba University Hospital, Sfax, Tunisia

**Keywords:** Pinna, hematoma, trauma

## Image in medicine

Auricular hematoma is a frequent complication of blunt trauma to the auricle. These are usually caused by sports such as boxing and wrestling. Hematoma formation is within the space between perichondrium and cartilage on the anterior ear. Because the blood supply to cartilage is dependent on the perichondrium, infection and cartilaginous necrosis often complicate untreated auricular hematomas. If auricular hematoma is not treated properly, it can progress to abscess formation, chronic scarring and fibrous organization with ear deformity “cauliflower” due to a stimulation of mesenchymal cells in the perichondrion to produce a new cartilage. After incision and evacuation of the fluid, a pressure dressings is traditionally applied to prevent reaccumulation in the resultant dead space. Optimal immobilization of the skin over underlying cartilage for 7-10 days prevent the reaccumulation of fluid with subsequent thickening and fibrosis. We report a case of a 40-year-old football coach presented to the Emergency Department with a painless right ear swelling occurred after auricular trauma during training. The clinical examination showed a right fluctuant non-inflammatory swelling of the lateral face of the pinna. Otoscopy and retroauricular region were normal. The needle puncture brought a serohematic fluid and the diagnosis auricular hematoma was made. The collection was drained via a double anterior incision under local anesthesia and a tie through dressing was used. After 10 days, the sutures and tie through dressing were removed. One year after the operation, the ear appeared almost normal.

**Figure 1:**
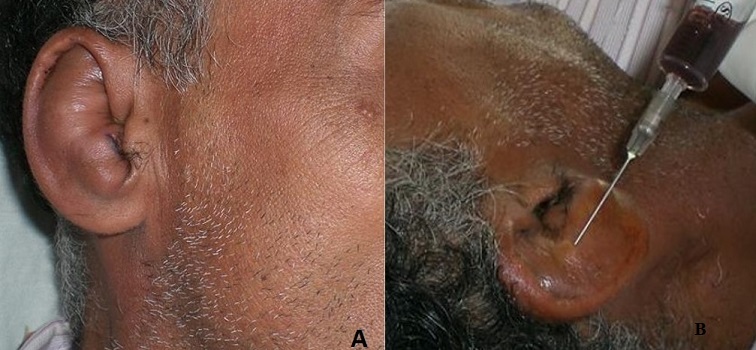
(A) the clinical examination shows a right fluctuant non-inflammatory swelling of the lateral face of the pinna; (B) the needle puncture brought a serohematic fluid

